# Efficacy in the treatment of malaria by *Plasmodium vivax* in Oiapoque, Brazil, on the border with French Guiana: the importance of control over external factors

**DOI:** 10.1186/s12936-015-0925-7

**Published:** 2015-10-09

**Authors:** Margarete do Socorro M. Gomes, José Luiz F. Vieira, Ricardo L. D. Machado, Mathieu Nacher, Aurélia Stefani, Lise Musset, Eric Legrand, Rubens A. O. Menezes, Aldo A. P. Júnior, Ana P. M. Sousa, Vanja S. C. D`Almeida Couto, Álvaro A. R. D’Almeida Couto

**Affiliations:** Central Laboratory of Public Health of Amapá (Laboratório Central de Saúde Pública do Amapá - LACEN-AP), Macapá, Amapá Brazil; Federal University of Pará (Universidade Federal do Pará - UFPA), Belém, Pará Brazil; ‘Evandro Chagas’ Institute (Instituto Evandro Chagas), Belém, Pará Brazil; Université des Antilles et de la Guyane, Cayenne, French Guiana; Centre d’Investigation Clinique – Epidémiologie Clinique Antilles-Guyane (CIC-EC INSERM CIE 802), Cayenne General Hospital, Cayenne, French Guiana; Institut Pasteur de la Guyane, Cayenne, French Guiana; Federal University of Amapá (Universidade Federal do Amapá - UNIFAP), Macapá, Amapá Brazil; Amapá State Health Department (Secretaria de Estado da Saúde do Amapá – SESA), Macapá, Amapá Brazil

**Keywords:** Malaria, *Plasmodium vivax*, Anti-malarials, Treatment efficacy, Brazil-French Guiana border, External factors, Treatment failure

## Abstract

**Background:**

*Plasmodium vivax* malaria is an important public health issue in the Amazon region, and it accounts for approximately 84 % of cases of the disease. Migration across the border between Brazil and French Guiana contributes to the maintenance of the disease. The aim of this study was to evaluate the therapeutic and parasitological responses of patients with *P. vivax* malaria treated with chloroquine and primaquine in the socio-environmental context of cross-border interactions between Brazil and French Guiana. The factors controlled were diagnostic agreement, adherence, adjustment of primaquine doses for patient weight, and quality of the drugs used.

**Methods:**

A prospective study was conducted in 2011 with 103 individuals aged 10–60 years with a positive diagnosis of *P. vivax* treated with chloroquine (10 mg base/kg on the first day, followed by 7.5 mg/kg on the second and third days) and primaquine for 7 days, who were followed for 28 days. The primaquine doses were adjusted for the patients’ weight. A number of factors were determined: epidemiological characteristics, origin of patients, signs and symptoms, initial parasitaemia and parasitaemia clearance time, blood concentrations of chloroquine and primaquine, quality of anti-malarial drugs and diagnostic agreement.

**Results:**

Ninety-five patients were followed for 28 days. There was a 100 % agreement in microscopic diagnosis between field laboratory and reference centre. The adhesion to the treatment was 100 %. Of these patients, 32.6 % received a weight-adjusted dose of primaquine. The chloroquine and primaquine tablets were consistent with the optimal quality limits for human consumption. The investigated patients achieved optimal blood exposure to anti-malarial drugs. The parasitological and therapeutic response was adequate in 99.0 % of cases.

**Conclusions:**

In the municipality of Oiapoque, the therapeutic regime used for the treatment of *P. vivax* malaria using chloroquine combined with primaquine remains effective, when external factors are controlled, such as the quality of anti-malarial drugs, the adhesion to the treatment prescribed, the correct diagnostic and the adjustment of primaquine dose for patient body weight.

## Background

*Plasmodium vivax* malaria is an important public health issue in the Amazon region, and it accounts for approximately 84 % of malaria cases [1]. Environmental characteristics, such as climate and hydrography, which are associated with movement of the *Anopheles* vector, contribute to maintenance of transmission of this disease in this area, which is considered as hypo- to meso-endemic, unstable and with annual seasonal fluctuations [1, 2]. The disease does not occur uniformly across the region, as locations with different levels of transmission have been observed [1, 2]. In the municipality of Oiapoque, which is located in the state of Amapá-AP in the far northern Brazilian Amazon and bordering French Guiana, the disease shows peculiar characteristics due to frequent cross-border migration. The municipality is at high risk of transmission, with approximately 5000 cases reported annually and a predominance of infections with *P. vivax,* which represent 82 % of cases [1].

Early diagnosis and timely and appropriate treatment play a central role in disease control because both prevent the occurrence of severe cases and consequently death as well as in eliminating the sources of infection for mosquitoes [3, 4]. The treatment of choice for *P. vivax* consists of chloroquine for 3 days combined with primaquine diphosphate for 7 days, and the second choice is primaquine for 14 days with the same dose of chloroquine [3, 4]. In Brazil, the national malaria control programme (*Programa Nacional de Controle da Malária* (PNCM)) provides guidelines for treatment and distribute anti-malarial drugs with no costs [4].

The variability of *Plasmodium* response to anti-malarial drugs limits therapeutic success. Parasites that appear at diagnosis (D0) and are not detected on the 28th day are considered sensitive [3]. Treatment failure may result from resistance of *P. vivax* strains circulating in a given endemic area in addition to other factors [5–7]. Factors intrinsic to the host, the parasite and the anti-malarial drugs, as well as their interactions, contribute to treatment failure. Such factors include intra- and inter-racial variability of the pharmacokinetic profiles of anti-malarial drugs, quality of anti-malarial drugs, accuracy in identifying the *Plasmodium* species, and exposure to low concentrations of anti-malarial drugs resulting from low quality of drugs, poor adherence to the prescribed regimen, vomiting and others gastrointestinal disorders, and finally, the adjustment of primaquine dose as a function of patient body weight [6–9].

Several studies have evaluated the efficacy of chloroquine plus primaquine for *P. vivax* in Brazil and in other countries of South America; however, the use of non-standardized protocols makes it difficult to compare the percentages of treatment failure [10–15]. Additionally, few studies have assessed in an integrated way the factors that are responsible for treatment failure but not directly related to resistance of the parasite or the host immune response [16–18]. Thus, this study aimed to evaluate the therapeutic and parasitological response of patients with vivax malaria treated with chloroquine and primaquine in the socio-environmental context of cross-border interactions between Brazil and French Guiana by considering adherence, concordance of diagnosis, primaquine dose adjustment for patient weight and quality of the anti-malarial drugs.

## Methods

### Study area

The study was conducted in the municipality of Oiapoque-AP in the Amazon region of far northern Brazil. Oiapoque shares an international border with French Guiana. Oiapoque is situed at sea level and has an area of 22.625 Km^2^. The annual average humidity ranges from 75 to 85 %. There are two seasons: rainy season (January–June), with a rainfall average of 1800 mm^3^ and dry season (July–December), with a rainfall average of 500 mm^3^. The annual average temperature ranges from 23 to 32 °C. The resident population is 20,509 inhabitants and fluctuates by 10,000–12,000 individuals. The annual parasite index (API) was 246/1000 population in 2011 [01].

### Patients

Individuals of both genders aged 16–60 years who spontaneously searched for public health care services in Oiapoque with signs and symptoms suggestive of malaria, from August to December 2011, and with a positive thick blood smear for vivax malaria were included in the study. Individuals with chronic and infectious concomitant disease, patients with mixed, severe or complicated malaria, pregnant women, indigenous people, patients with reports of prior adverse reactions to the drugs and those who disagreed or did not sign the consent form were excluded.

### Quality control of chloroquine diphosphate and primaquine diphosphate tablets

The drugs used were supplied by the Coordination of Pharmaceutical Assistance of Amapá (*Coordenadoria de Assistência Farmacêutica do Amapá*). Prior to use, the validity, concentration and lots of all drugs were carefully checked. Moreover, physicochemical analyses were performed prior to use, which consisted of determining the weight of the tablets according to the general methods described in the Brazilian Pharmacopoeia [19]. Next, dissolution tests and determination of the active ingredient content were performed as described for each drug in the Brazilian Pharmacopoeia [20]. In the dissolution test, a tolerance of 70 % of chloroquine diphosphate and 80 % of primaquine diphosphate (relative to the amount declared on the label) dissolving in 45 min was adopted. The values obtained in the test for determining the active ingredient content of the tablets were required to be between 93.0 and 107.0 % of the labelled value.

### Clinical assessment

Clinical assessments were carried out after confirmation of vivax malaria, before treatment with anti-malarials (D0) and after one, three, seven, 14, 21, and 28 days. The typical signs and symptoms of malaria as well as non-specific symptoms were classified as ‘present’ or ‘absent’ [3, 4]. Patients included in the study were carefully informed of the need for a replacement dose in case of vomiting less than 30 min after ingestion of the drug, and in case of heavy and prolonged diarrhoea, they were asked to immediately contact the healthcare team.

### Therapeutic regime

The first-line regimen used to treat vivax malaria consisted of chloroquine diphosphate (Farmaguinhos Laboratory –Fundação Oswaldo Cruz/Rio de Janeiro, Brazil) at doses of 10 mg base/kg on the first day followed by 7.5 mg/kg on the second and third days, corresponding to a total dose of 25 mg/kg, combined with primaquine diphosphate (Farmaguinhos Laboratory – Fundação Oswaldo Cruz/Rio de Janeiro, Brazil) at a dose of 0.50 kg base/kg for 7 days, corresponding to a total dose of 3.0–3.5 mg/kg. The dose of primaquine was adjusted for weight for patients weighing 70 kg or more as shown in Table 1 [3, 4]. The patients were weighed on the day of inclusion in the study using a previously calibrated balance with digital scale. A patient who exhibited treatment failure with chloroquine + primaquine received a regimen of weekly chloroquine for 12 weeks [3, 4].

**Table 1:** Adjustment of primaquine dose and time of administration for patients weighing more than 70 kg

Weight class (kg)	Total dose of primaquine (mg)	Time of administration (days) Short regimen (30 mg/day)
70–79	240	8
80–89	272	9
90–99	304	10
100–109	336	11
110–120	368	12

*Source*: Ministry of Health (Ministério da Saúde), Brazil [4]

### Adherence to treatment

The adherence by patients to treatment was evaluated during follow-up clinical interviews, as well as by pill counting performed daily at home of patients. Subsequently, these values were compared with blood anti-malarial drug concentrations [9, 21, 22].

### Parasite density and parasitaemia clearance time

The thick smear was stained with Giemsa following Walker’s method and examined under a light microscope. Parasitaemia was determined by counting the parasites in 100 microscopic fields (750 times), and this value was multiplied by five, with the result expressed as parasites per cubic millimetre of blood. Smears were considered negative (i.e., absence of parasitaemia) when, after examining at least 200 microscopic fields, asexual forms of *P. vivax* were not found. Three levels of parasitaemia were defined: low, intermediate, and high, which corresponded to up to 15,000, 15,001–60,000, and equal to or greater than 60,001 parasites per mm^3^ of blood, respectively. The parasitaemia for inclusion in the study ranged from 200 to 100,000 parasite per mm^3^ of blood. The parasitaemia clearance time (PCT) of the patients was checked every 24 h using thick-smear slides on days 1, 2, 3, 4 5, and 7. Values are expressed as the means and standard deviation [23, 24].

### Diagnostic agreement

All positive slides were reviewed in the Central Laboratory of Public Health of Amapá (*Laboratório Central de Saúde Pública do Amapá*—LACEN-Amapá) reference laboratory.

### Blood samples

Blood samples (approximate volume of 20 μL) were collected during clinical assessments using finger-prick lancets. Samples were then mounted on glass slides to obtain thick blood smears [25]. For the dosage of the anti-malarials, venous blood samples were collected in EDTA anticoagulant on days 0, 3, 14, 21, and 28, applied to filter paper (Whatman 17Chr,^®^ Pennsylvania, USA), and dried at room temperature for 4 h. Then, the filter paper was placed in a paper envelope.

### Determination of blood concentrations of chloroquine and primaquine

Chloroquine and primaquine were measured in order to assess the adherence. A reverse-phase high-performance liquid chromatography system was used for the measurement of both drugs. The chromatographic conditions for determination of primaquine were as follows: UV detection: 254 nm; flow rate: 1 mL/min; column: RP-8.15 cm, 5 μm and 4.6 mm internal diameter (Waters, Saint Quentin-en-Yvelines, France); mobile phase: acetonitrile:methanol:water (30:26:95, v/v); pH 5; injection volume: 50 mL. The validated analytical procedure for the determination of primaquine concentrations showed that the method was linear from 10 to 900 ng/mL. The mean intra- and inter-day coefficients of variation were 8.3 and 12.1 %, respectively. The limit of detection was 5 ng/mL and the limit of quantification was 10 ng/mL. The mean retention time of primaquine was 8.8 min and of quinidine (internal standard) was 7.0 min. The mean recovery of primaquine was 90.3 %. The stability of samples supplemented with primaquine was 120 days under the conditions described above [26].

For chloroquine, the chromatographic conditions were as follows: column: C-8, Xterra 4.6 × 150 mm in size, 5-μm particles, reverse phase RP18; pre-column: Xterra, RP18 3.9 × 20 mm, 5-μm particles; mobile phase: 40 % acetonitrile, 60 % (1 % triethylamine in water); pH 10.5, flow rate: 1.2 mL/min; UV detection: 333 nm; excitation: 330 nm; temperature: 25 °C; injection volume: 50 μL. The mean retention time for chloroquine was 20.0 min and for quinine (internal standard) was 6.0 min. The limit of detection was 25 ng/mL and the limit of quantification was 38 ng/mL. The method was linear from 38 to 250 ng/mL. The mean recovery was 87 %. The intra- and inter-day coefficients of variation were 10.5 and 13 %, respectively. The stability of samples supplemented with chloroquine was 90 days [27].

### Classification of parasitological and therapeutic responses

The World Health Organization classification criteria for parasitological response in areas with intense transmission was adopted, with sensitive (S) or resistant types I, II and III [3]. Parasitological cure was defined as the disappearance of asexual parasitaemia after beginning a specific treatment and its absence during the following 28 days, and treatment failure was evidenced by the recurrence of parasitaemia during this period. Resistance to chloroquine was identified based on the recurrence of parasitaemia in the presence of minimum effective concentrations of chloroquine (CQ) and desethylchloroquine (DCQ) of 100 ng/mL in whole blood during follow-up, regardless of the source of recurrence, i.e., resurgence, re-infections and relapses [6, 7].

### Sample size

The sample size was based on the World Health Organization protocol for studies of the efficacy of anti-malarial drugs in the Americas, specifically of the efficacy of chloroquine in the treatment of vivax malaria. A minimum of 81 patients was required, but the study included 103 patients, with 95 who were fully monitored [3, 28].

### Statistical analysis

Samples are expressed as the means (SD) and as median and range when required. The Fisher’s exact, Chi square and G tests were used to compare the variables: gender, age, origin of cases, and signs and symptoms of the disease. The correlation between parasitaemia and the presence of signs and symptoms of infection was determined by the Spearman’s correlation coefficient (s). The Mann–Whitney test was used to compare parasitaemia clearance times. The accepted level of significance was 5 %.

### Ethics

The study was approved by the Research Ethics Committee of the SEAMA School under protocol number CEP/SEAMA No. 079/08 of 11/27/2008 as part of the main project. Candidate participants were only included in the study after receiving clarification and providing written agreement to participate in the study.

## Results

A total of 103 patients with laboratory diagnosis of vivax malaria were initially included in the study. Of these, 95 completed the follow-up. Six participants were excluded due to loss to follow-up, because they went away of Oiapoque. Another two patients were excluded due to concurrent infections with *Plasmodium falciparum*, which was diagnosed during the first week of follow-up.

### Epidemiological characteristics and origin of cases

The majority of patients were males (67 %). The mean age was 30 (±12) years, ranging from 16 to 60 years. The age group most affected by the disease was 20–29 years followed by 30–39 years for both genders. A total of 19 % of cases corresponded to primary infection. Reports of previous infection were divided into one, two and more than four, corresponding to 2, 15 and 64 % of cases, respectively. Autochthonous cases in Oiapoque represented 69 % (*p* = 0.0002). Of cases originating in French Guiana, the illegal gold mining site of ‘Sikini’ was the most commonly cited with 21.9 % of cases (*p* < 0.0001).

### Quality of chloroquine diphosphate and primaquine diphosphate tablets

The mean weight of the chloroquine diphosphate tablets was 412 mg, with a unit-to-unit variation of −2.50 to 3.30 %, while the mean weight of the primaquine diphosphate tablets was 128 mg, with a unit-to-unit variation of −5.30 to 4.25 %. No tablet unit was outside the weight range recommended in the Brazilian Pharmacopoeia, i.e., between −7.5 and 7.5 %. The dissolution test for chloroquine diphosphate tablets showed a release rate of 78 % (± 2.4) in 45 min, which is above the upper limit of 70 %. The primaquine diphosphate tablets exhibited a release rate of 85 % (±3.2) in 45 min, which was also above the minimal tolerance value of 80 %. The mean chloroquine diphosphate content in the tablets was 98.31 (2.11) %, ranging from 96.33 to 103.45 % of the labelled value. The mean primaquine diphosphate content in the tablets was 99.87 (2.28) %, ranging from 98.12 to 101.13 %, i.e., within the acceptable range of 93.0–107.0 % of the labelled value.

### Clinical findings

Several clinical symptoms were reported, including fever (88 %), chills and headache (86 %), sweating (83 %), myalgia (43 %), and nausea (56 %). Despite fever being the most frequent clinical manifestation, it was not the initial complaint in 12 % of patients. Other symptoms, such as headache, myalgia and nausea were initially mentioned. The classic triad of malarial symptoms (fever, chills and headache) was reported in 46.7 % of cases. There was a significant correlation between parasitaemia and the presence of signs and symptoms of infection (*p* < 0.0001).

### Dose adjustment of primaquine

The dose of primaquine was adjusted in 32.6 % of patients weighing 70 kg or more by adding two 15-mg tablets on each day, which is equivalent to 0.30 mg/day. In this group, the total dose of primaquine ranged from 240 to 304 mg. The duration of administration was increased from 8 to 10 days to reach the total dose of primaquine. The adjustment of doses did not alter blood concentrations of primaquine (*Z* = 1.5195, *p* = 0.0643). It is noteworthy that the relapsed patient did not require adjustment, as the patient weighed 55 kg.

### Adherence to treatment

The pill count during the clinical assessments carried out during treatment to evaluate the adherence indicated a rate of 100 % adherence to treatment. This finding was corroborated by anti-malarial blood concentrations on follow-up assessment days.

### Parasite density and parasitaemia clearance time

The geometric mean of parasitaemia was 1168 (±3.32) parasites/mm^3^ on D0. The parasitaemia decreased beginning on D2 and remained negative until D28 (Table 2), except for one patient who had 400 parasites/mm^3^ on D28. The parasitaemia on D0 was low in 90 patients and intermediate in five patients (*p* < 0.0001). There were no cases of high parasitaemia.

**Table 2:** Parasite density in patients with *Plasmodium vivax* malaria in Oiapoque/AP

Parasites: asexual form	Days of follow-up					
	D0	D3	D7	D14	D21	D28
Geometric mean (V/mm^3^)	1167.86	40.33	0	0	0	0
Geometric standard deviation	±3.32	±4.07	0	0	0	0
Minimum (parasites/mm^3^)	200	5	0	0	0	0
Maximum (parasites/mm^3^)	36000	350	0	0	0	0
Relapsed patient	3000	350	0	0	0	400
Number of patients	95	19*	0	0	0	01**

*Source*: Primary data, 2013

* Patients who had positive parasitaemia on D3

** Patient who had positive parasitaemia on D28

The parasitaemia clearance time ranged from 48 to 120 h. All patients were negative for parasitaemia during the first week of treatment. Of these, 80 % were negative on D3 and the rest during the first week (D0–D7). Patients with an intermediate parasite density showed a longer parasitaemia clearance time compared to those with low parasite density (*p* = 0.0284).

### Diagnostic agreement

Comparison of the diagnoses by thick smears performed in Oiapoque and during the quality control process by LACEN-AP indicated 100 % diagnostic agreement.

### Blood concentrations of chloroquine and primaquine

Blood concentrations of chloroquine and primaquine during follow-up visits are shown in Table 3. The whole blood chloroquine concentrations during follow-up were greater than 100 ng/mL, including the patient who relapsed with positive parasitaemia on day 28 of follow-up.

**Table 3:** Blood concentrations of chloroquine diphosphate and primaquine diphosphate in ng/mL

Description	Chloroquine diphosphate in ng/mL				Primaquine diphosphate in ng/mL			
Days of follow up	D0	D1	D3	D28	D0	D1	D3	D28
Number of patients	95	95	95	1	95	95	95	1
Mean level	ND	461.1	119.6	–	ND	32.47	50.8	–
Standard deviation	ND	224.9	495.9	–	ND	17.63	22.1	–
Minimum level	ND	209	286	–	ND	13.6	18.0	–
Maximum level	ND	992	2213	–	ND	99.5	98.3	–
Relapsed patient	ND	324	1120	210	ND	58.9	26.2	ND

*Source*: Primary Data, 2013

*ND* Not detected

### Classification of parasitological and therapeutic responses

The therapeutic response was considered appropriate in 94 patients (99 %) with complete disappearance of symptoms and clearance of parasites, which was classified as sensitive (S). One participant (1.0 %) had late treatment failure, with a parasitological response classified as resistance type RI (Table 4). This patient was treated with chloroquine for 12 weeks taking care to ensure correct patient adherence to conventional treatment, following the recommendation of Brazilian Health Office.

**Table 4:** Classification of parasitological and therapeutic responses

Clinical response	ACR		LTF		ETF				
Parasitological response	S		RI		RII		RIII		Total
Regimen	Nº	%	Nº	%	Nº	%	Nº	%	
Chloroquine + primaquine	94	99.0	01	1.0	–	–	–	–	95

Primary source

*ACR* adequate clinical response, *LTF* late treatment failure, *ETF* early treatment failure, *S* sensitive, *RI* resistance type I, *RII* resistance type II, *RIII* resistance type III, *N* absolute number

## Discussion

Malaria on the French Guiana-Brazilian border has been linked to high population mobility towards the French gold mines, where its incidence is high [29]. Although most cases are autochthonous to Oiapoque, intense cross-border migration precludes confirmation of the exact location of infection. Indeed, the predominance of cases in adult males in the economically active age groups show the importance of illegal gold mining in areas of high occurrence of the disease in French Guiana, such as the Sikini gold mine.

Low to moderate parasitaemia is common in *P. vivax* infections in the Brazilian Amazon and may have been responsible for the classic triad of the disease being diagnosed in only half of patients, despite reports of severe cases with low parasitaemia and the influence of host immune status, comorbidities, strain virulence, and others in the initial clinical manifestations. In addition, the parasitaemia clearance time observed in the study resembled that of other endemic areas in which the sensitivity of *P. vivax* to chloroquine is maintained [12–15, 30].

The mean blood concentrations of chloroquine during follow-up visits corroborate the findings in other population groups, including the Brazilian Amazon, under the same treatment regimen [15, 31, 32]. Similarly, the mean blood concentrations of primaquine during treatment resembled those previously reported in Indian patients with vivax malaria under the same therapeutic regimen [33].

The adequate parasitological response at the end of clinical-laboratory follow-up showed the therapeutic efficacy of the standard treatment in 99 % of cases, confirming previous findings in patients treated with the same therapeutic regimen in Belém, in the Brazilian state of Para [34]. However, the data show only high effectiveness of chloroquine against *P. vivax* blood stages but do not ensure a radical cure, as in Brazil relapse can occur up to 6 months following the completion of treatment [35, 36]. In addition, the adequate parasitological response of patients with recurrent vivax malaria corroborates the efficacy of treatment. High rates of recurrent infection are commonly reported in areas of endemic transmission of the disease and have been associated with a new infection or relapse of hypnozoites. Studies in the Brazilian Amazon have reported decreased efficacy of these drugs for the treatment of *P. vivax* [10, 12, 15, 35, 36]. However, there are several difficulties to follows the standardized guidelines proposed by WHO for evaluation of the efficacy of anti-malarial drugs in Brazilian Amazon basin either by its continental dimensions or by the larger border with other Amazon countries with their own police of diagnosis and treatment of the disease [2, 9–12, 15, 16]. These limitations impose methodological differences, such as the length of treatment, dose adjustments of primaquine as a function of patient weight, dose administered, and the no assessment of anti-malarial drugs exposure, hinder comparisons of studies of the therapeutic efficacy of the chloroquine and primaquine combination against *P. vivax* [2, 9–12, 15, 16]. As an example, epidemiological records of the Malaria Epidemiological Surveillance Information System (*Sistema de Informação de Vigilância Epidemiológica*—SIVEP-Malária)/MS in Oiapoque indicated that 14.2 and 13.5 % of cure verification slides were positive in 2009 and 2010, respectively. However, one should consider that the examination in this study was conducted on day 28 after beginning treatment, as opposed to the 60 days recommended by the Ministry of Health after treatment for *P. vivax*.

The control of factors responsible by treatment failures in vivax malaria evaluated in the current study, such as the adjustment of the dose of primaquine for patient weight, the intrinsic quality of the drugs, and the adherence of all patients to the prescribed treatment assessed through interviews, pill counts and confirmation of drug exposure by the concentrations of anti-malarial drugs in blood may have contributed to the high effectiveness of the standard treatment. In addition, the efficiency of disease control policies in Brazil, based on early and correct diagnosis, as well as in the timely and appropriate treatment of cases, may have also contributed to these findings, which was corroborated by the agreement of thick blood smear diagnoses between different treatment centres, as well as by the absence of anti-malarial drugs in pre-dose samples [4].

Late treatment failure in one patient could be classified as resistance type RI. The plasma concentration of chloroquine at D28 was greater than 100 ng/mL, indicating adequate drug exposure as well as the presence of blood strains of *P. vivax* at concentrations above the minimum inhibitory concentration for chloroquine-sensitive strains. The treatment failure could be explained by the fact that there are different parasite genotypes circulating in the region [6, 7, 25, 37]. In addition, it is important to highlight that the only patient with treatment failure shows concentrations of primaquine on day 3 approximately 50 % lower than on day 1, while in the remaining levels on day 3 were higher than in day 1. This finding could be useful to explain the treatment failure see in the current study.

## Conclusion

In the municipality of Oiapoque the treatment regimen of chloroquine combined with primaquine used for the treatment of vivax malaria remains effective. In addition, circulating *P. vivax* isolates have no significant resistance profile to the currently recommended treatment. The control over external factors, such as the dose adjustment of primaquine based on the weight of the patient, the quality of anti-malarial drugs and adherence to treatment, contributed to the high effectiveness of the standard treatment found in the study.
